# The Equivalence between Virtual and Real Feared Stimuli in a Phobic Adult Sample: A Neuroimaging Study

**DOI:** 10.3390/jcm8122139

**Published:** 2019-12-04

**Authors:** Wenceslao Peñate, Francisco Rivero, Conrado Viña, Manuel Herrero, Moisés Betancort, Juan De la Fuente, Yolanda Álvarez-Pérez, Ascensión Fumero

**Affiliations:** 1 Universidad de La Laguna, 38200 La Laguna, Spain; wpenate@ull.edu.es (W.P.); friverop@ull.edu.es (F.R.); cmvinalo@ull.edu.es (C.V.); mherrero@ull.edu.es (M.H.); moibemo@ull.edu.es (M.B.); 2 Servicio Canario de la Salud, 38004 S.C. Tenerife, Spain; jafuente@ull.es (J.D.l.F.); yolanda.alvarezperez@sescs.es (Y.Á.-P.)

**Keywords:** virtual reality, real phobic images, anxiety disorders, specific phobia, fMRI, neuroimaging

## Abstract

The clinical use of virtual reality (VR) has proven its efficacy, especially when used as an exposure technique. A prominent property of VR’s utility is its equivalence with the reality it represents. In this study, we explored this equivalence in a clinical context using neuroimaging. A sample of 32 adults with specific phobias (i.e., to cockroaches, spiders, or lizards) was divided into two groups: One was exposed to phobic stimuli using VR and the other was exposed to real phobic images (RI). We used brain activations as a dependent measure, focusing specifically on brain areas usually associated with fear processing. Whole-brain analysis detected higher activations for RI in the hippocampus, occipital, and calcarine areas. A specific analysis of the amygdala and insula also detected higher activations and extensions in response to RI, but VR stimuli also activated those areas in a significant manner. These results suggest that even in those cases where RI stimuli activate all of the brain’s fear-processing circuits, VR stimuli do so as well. This implies that VR can be useful as an exposure technique similar to RI and applied as more than a mere training mechanism.

## 1. Introduction

The use of virtual reality (VR) as a tool for psychological treatment has grown since it was first employed [1] as an exposure procedure for the treatment of phobic disorders. Since then, many studies have been conducted with VR as the main therapeutic resource, which has maintained interest in this topic over the years [2]. Although VR has been employed in several therapeutic approaches, it most often takes the form of “virtual reality exposure therapy” (VRET) [3].

The efficacy and efficiency of VRET have been proven in a considerable number of clinical trials and experimental designs, as shown by several systematic reviews and meta-analyses [3,4,5,6]. There are several reasons for this efficacy: VR is an intermediate step in graduated exposure, a safe condition in which to train patients to cope with real stimuli. VR also enhances exposure when VRET is combined with in vivo exposure [5,6,7,8,9,10]. Interestingly, two processes in the functioning of VR can be inferred from these explanations: VR accurately represents real stimuli (exposure process [8]), and VR is an opportunity to cope with a distressing stimulus (training process [11,12]).

The efficacy of VRET has a prerequisite related to the physical properties of the virtual scenarios used. The key concept here is immersive technology. This concept is related to VR’s property of “enveloping” participants and making them feel as if they are “actually there”. There are several definitions of immersive technology [13]. These definitions share the idea that the more similar a virtual context is to a real one, the greater its immersive power. Technical characteristics (e.g., environments, distinctness, movements) and the type of presentation (i.e., 3D) are physical attributes that can shape a more immersive and consequently more effective VR [14]. Implicitly, the fact that VR scenarios are “immersive” also means that they are processed similarly to real ones. However, this property of sensation of presence, being a prerequisite, is not sufficient to explain the efficacy of VRET [15]. This efficacy seems to be supported in the property of VR to stimulate a brain representation to create an embodied simulation of the body in the world, including main informational processes: Visceral/autonomic, sensory, and motor information [15]. As a consequence, observed brain activations as a function of VRET need to reflect this embodied simulation.

Functional magnetic resonance imaging (fMRI) studies have shed light on how patients with phobias process phobic stimuli. According to systematic reviews and meta-analyses [16,17,18,19,20], the presence of phobic stimuli is associated with greater activation of the left amygdala and insular cortex than of other brain areas. Other structures involved in phobic responses are the fusiform gyrus, the left dorsolateral prefrontal cortex, and the left cingulate cortex. Compared to the limbic areas, frontal areas have been found to be less consistent and less stable in processing phobic stimuli. These findings are congruent with the existence of a dual-route functional network in processing feared stimuli [21,22,23,24]: Wave1, a short/unconscious route that involves a direct link between the thalamus and the amygdala; and Wave2, a long/conscious route that involves the thalamus-sensory and cortex-entorhinal cortex–hippocampus–subiculum–amygdala.

Given that therapeutic exposure is related to how patients process phobic stimuli, the activation of these routes can have direct implications for exposure efficacy: Conscious routes imply more complex processing and a better opportunity for patients to change the significance of a phobic stimulus and develop a more adaptive response. In this regard, the present study was aimed at testing whether exposure to virtual phobic stimuli in a group of patients with specific phobias (i.e., to small animals) could activate the same brain regions as exposure to real image stimuli. We also intended to test whether virtual stimuli could also facilitate the activation of the conscious Wave2 route in processing phobic stimuli (as real image stimuli do) and to consider the implications of these data for the efficacy of exposure techniques. Specifically, we planned to compare activity in empirically supported brain regions associated with phobic stimulus processing.

## 2. Materials and Methods

### 2.1. Participants

The sample was composed of 32 adults. There were 26 (real phobic images (RI): 40.6%; VR: 40.6%) female and 6 (RI: 9.4%; VR: 9.4%) male participants. Sixteen participants (mean age 35.25 years, SD 12.17) were exposed to real images of small animals (i.e., cockroaches, spiders, or lizards), and 16 participants (mean age 33.43 years, SD 10.26) watched films of virtual images of such animals. The phobic stimulus matched the individual’s phobia.

All participants were right-handed and had normal vision. The main inclusion criterion was being an adult with a diagnosis of specific phobia. The phobia had to be a primary psychological disorder and not be explained by another health condition. Other inclusion criteria for participants included not receiving any treatment for a specific phobia at the time of the study and not having any impediment to undergoing a magnetic resonance imaging session.

### 2.2. Instruments

The Composite International Diagnostic Interview (CIDI), Version 2.1 (WHO, Geneve, Switzerland) [25] was used to verify the diagnosis of phobia. The CIDI is a structured interview for major mental disorders according to the CIE-10 criteria [26]. For the purposes of this study, questions related to a specific phobia, social phobia, agoraphobia, and panic attacks were selected. Participants diagnosed with a specific small-animal phobia were included (F40.218; [26]).

The S–R Inventory of Anxiousness [27] is a 14-item inventory with a 5-point Likert-type scale that assesses physiological, cognitive, and behavioral anxiety symptoms associated with an anxiety-inducing situation. The phobic stimulus target is pointed out prior to the participant′s response. The inventory has shown high internal consistency (0.95) and adequate convergent validity [27,28]. For the current study, Cronbach’s alpha was 0.79.

The Beck Anxiety Inventory (BAI) [29] is a 21-item self-report instrument for assessing the severity of anxiety states. Participants are asked to rate the severity of each symptom using a 4-point Likert-type scale ranging from 0 (“Not at all”) to 3 (“Severely—I could barely stand it”). Total scores range from 0 to 63. Scores of 26–63 represent severe anxiety [29]. Cronbach’s alpha for the current study was high (0.93).

Hand preference was assessed with the Edinburgh Handedness Inventory (EHI) [30]. This inventory consists of ten items: Writing, drawing, throwing, using scissors, toothbrush, knife (without fork), spoon, broom, striking a match, and opening a box. Participants indicated the strength of their hand preference for each of the 10 items by putting one or two ticks in the appropriate column, or one tick in each column if they were indifferent about that item. The EHI provides a Laterality Quotient ranging from +100 (totally right-handed) to −100 (totally left-handed).

### 2.3. Design

A 2 × 2 factor design was used: The first independent variable was “stimulus format” with two levels (real images and virtual reality), and the second independent variable was “type of stimulus” (phobic and neutral stimulus).

The stimuli consisted of small animals in motion. These were filmed in 3D video for the real images. To control the presentation modality effects, 3D recorded movies were used as the models to create the virtual reality stimuli. The arousal properties of these virtual reality stimuli were tested by measuring activations of the brain regions of interest (ROIs) at the initial fMRI session. Because the virtual stimuli were directly related to each specific phobia (i.e., participants with a spider phobia received only spider stimuli), stimulus valence was not assessed. All participants were informed about the stimulus format (virtual or real images).

**Figure 1:**
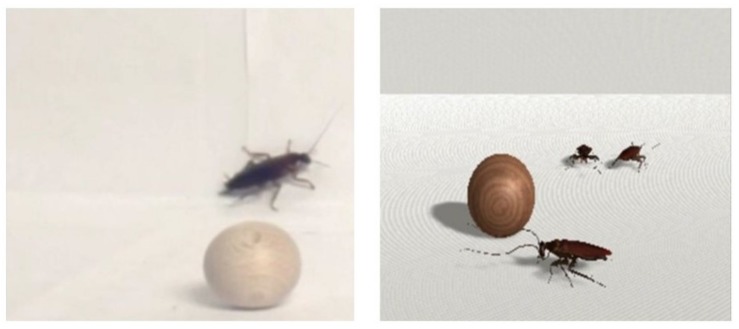
Example of the real image (RI) and virtual reality (VR) stimuli.

Both the real image and virtual reality formats included both phobic stimuli (i.e., cockroaches, spiders, or lizards) and neutral stimuli (i.e., wooden balls). All images were presented before an identical white background. Stimuli were presented in 3D virtual reality video format (VR group) and 3D real image video format (RI group). Figure 1 shows examples of the RI and VR stimuli.

Participants were randomly assigned (direct method) to one of two groups: One received the stimuli in virtual reality format (VR group) and the other received them as real images (RI group). Participants were exposed to two different conditions: Phobic stimuli and neutral stimuli (i.e., wooden balls). 

We used neuroimaging activations as dependent variables. The images were filtered for ROI, taking into account previous results with patients with phobias [16,17,18,19,20]. Nine regions were selected for both hemispheres: Amygdala, hippocampus, insula, fusiform gyrus, occipital cortex (inferior, medial, and superior), calcarine area, and thalamus. 

Stimuli were recorded in 3D and projected in the MRI scanner in stereoscopic 3D video using Visual Stim digital MRI-compatible 3D glasses (graphics card: GeForce 8600GT), (Resonance Technology Inc., Northridge, CA, US). We presented the stimuli using a block design. Each participant was randomly presented with 16 blocks of phobic images and 16 blocks of images of wooden balls. The duration of each block was 20 seconds.

### 2.4. Procedure

The study was conducted from April to July 2016. Phobic participants were recruited through various media (i.e., website, press, flyers, radio, TV, and newspapers). Next, an e-mail with the inventories was sent to possible participants. The initial diagnosis of specific phobia according to participants’ inventory scores was corroborated by a semistructured interview. Those who did not meet the inclusion criteria (or met the exclusion criteria) were excluded. In addition, due to the interference with the fMRI analysis, participants with nonremovable metal devices were excluded. Participants signed an informed consent form included in the study that had been approved by the Ethics Committee for Research and Animal Welfare of the University of La Laguna (CEIBA2012-0033). After their participation, subjects were entitled to receive as payment an eight-session free psychological treatment for specific animal phobia.

### 2.5. fMRI Data Acquisition

Functional MRI data were collected with a 3T General Electric Signa Excite scan (General Electric, Madrid, Spain). The BOLD signal was measured with an echo planar imaging sequence with 30 ms of echo time, 2000 ms of repetition time, 25.6 of field of view, and 75° of flip angle. The image dimensions were 64 × 64 × 32 mm with 4 × 4 × 4 mm voxel dimensions.

### 2.6. fMRI and Data Analysis

Brain images were analyzed with Statistical Parametric Mapping (SPM 12) software (London University College, London, UK). Preprocessing procedures included realigning, coregistering, segmenting (with forward deformation fields), normalizing (structural images with a 1 × 1 × 1 mm voxel size and functional images with a 4 × 4 × 4 mm voxel size), and smoothing (Gaussian Kernel of 8 mm, FWHM). Images were rendered and adjusted to the standard brain template of the Montreal Neurological Institute (MNI). 

The 2 × 2 factor design was tested with a two-way ANOVA to compare the main effects of image format and stimuli and the interaction effect between image format and stimuli on the whole-brain activation.

In addition, images were filtered for ROIs (amygdala, hippocampus, insula, fusiform gyrus, occipital cortex, calcarine area, and thalamus). All these ROIs were extracted from the WFU Pickatlas 3.0.5b (Radiology Informatic and Imaging Laboratory, Winston-Salem, NC, US) for SPM 12 with the Automated Anatomical Labeling (AAL2) brain atlas and Brodmann areas atlas.

The Family-Wise Error (*p* < 0.05 FWE corrected) correction was used. However, noncorrected probabilities were admitted when they were congruent with the biological model of phobias (but never higher than 0.001 uncorrected). The error was corrected considering that there was activation when the activated area was equal to or greater than a 3-voxel cluster with a voxel size of 4 × 4 × 4 mm.

## 3. Results

**Figure 2:**
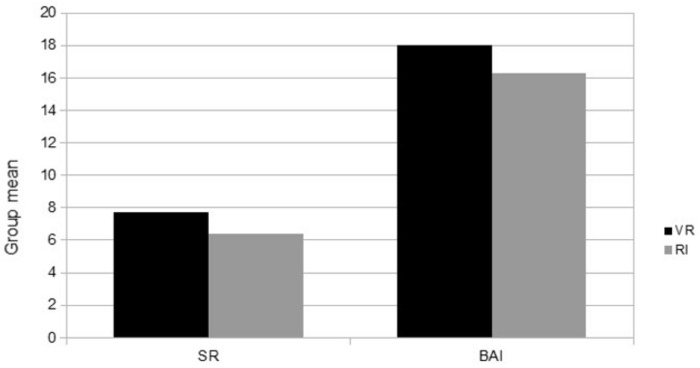
Anxiety measures.

An initial statistical analysis was performed to test the comparability between the VR group and RI group in anxiety measures (S–R and BAI scores). No significant differences (see Figure 2) were found between the two groups in these variables ((S–R: VR group M = 7.75, SD = 2.7; RI group M = 6.4, SD = 4.22) (BAI: VR group M = 18.0, SD = 14.14; RI group M = 16.3, SD = 11.95)).

**Figure 3:**
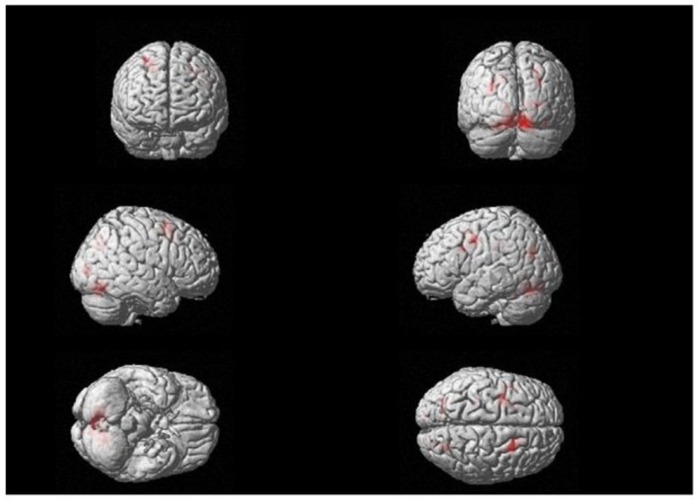
Whole-brain interaction effect. F (1.60) = 25.22, *p* < 0.05 (Family Wise Estimation (FWE)).

After that, a two-way ANOVA (whole-brain analysis) was conducted with image format (virtual, real) and type of stimulus (phobic, neutral) as independent variables. The whole-brain activations are shown in Figure 3. The interaction effect was significant (F (1.60) = 25.22, *p* < 0.05). This value was considered as the F score threshold. Moreover, an overall main effect was found for type of stimulus (F (1.60) = 26.78, *p* < 0.05). This main effect revealed a significant difference between phobic stimuli and neutral stimuli in brain activity: Fear-related stimuli generated higher brain activation than neutral stimuli. There were no differences in brain activity according to the image format factor (virtual vs. real phobic stimuli).

The following analyses were performed on the brain areas selected as ROIs. For exploratory reasons, a significance threshold of *p* ≤ 0.001, uncorrected *k* ≥ 3, was used to detect subtle changes in brain activation. In addition, to reduce the probability of false positive results, we set a contiguity threshold for cluster volumes of at least 20 voxels with a size of 2 × 2 × 2 mm [31] and did not consider clusters with a *Z* lower than 3.00. The fMRI comparisons between the RI group and the VR group in ROI brain areas are summarized in Table 1. These results showed significantly higher activations with real images in the hippocampus (R: F(1.60) = 25.77, *p* < 0.001; L: F(1.60) = 23.74, *p* < 0.001), fusiform gyrus (F(1.60) = 48.44, *p* < 0.000), bilateral middle occipital cortex (R: F(1.60) = 41.25, *p* < 0.000; L: F(1.60) = 46.09, p < 0.000), bilateral superior occipital cortex (R: F(1.60) = 44.78, *p* < 0.000; L: F(1.60) = 37.34, *p* < 0.00), and bilateral calcarine area (R: F(1.60) = 32.07, *p* < 0.01; L: F(1.60) = 29.53, *p* < 0.01). No differences were observed in the amygdala, insula, bilateral inferior occipital cortex, or thalamus in RI compared to VR.

**Figure 4:**
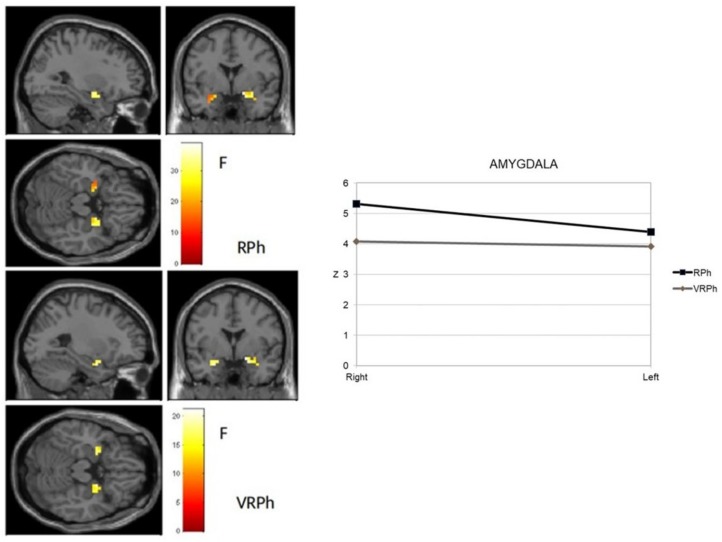
Amygdala activation of phobic stimuli processing (virtual and real format) in an adult sample with a specific phobia. RPh: Phobic real image; VRPh: Phobic virtual reality image.

A new and specific ROI analysis was performed separately for the amygdala and insula as two brain areas usually associated with the processing of anxiety-related stimuli, taking virtual vs. real image phobic stimuli as an independent variable. Figure 4 shows the activation observed in the amygdala. The response to real image stimuli was significant in a cluster in both the right amygdala (18, 0, −14) and left amygdala (−26, −4, −22), which both exhibited intense activity (t mean = 6.21; t SD = 0.20 right side, and t mean = 4.98; t SD = 0.54 left side). For VR stimuli, significant activity occurred in both the right (18, 0, −14) and left clusters (−26, 0, −22), which showed similar intensity (t mean = 4.59; t SD = 0.27 right side, and t mean = 4.40; t SD = 0.19 left side). These data revealed that stimulus processing was greater and more extensive in the real image format than in virtual reality.

**Figure 5:**
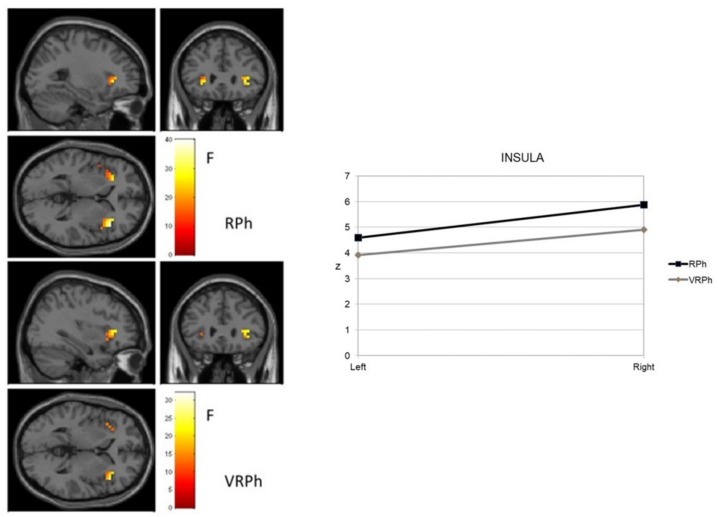
Insula activation of phobic stimuli processing (virtual and real format) in an adult sample with specific phobia.

Figure 5 shows activation of the insula. A similar activation was found in the right insula (42, 24, 2) with both real and virtual images. Yet, in the left insula, real images were associated with greater intensity (*Z* = 7.10; *p* < 0.000; *Z* = 6.22; *p* < 0.000; *Z* = 5.47; *p* < 0.000; *Z* = 5.45; *p* < 0.000) and extension (42, 24, 2; −46, 12, −6; −30, −28, 22; 38, −28, 22). These data suggest that fear-related (compared to neutral) images preferentially activated many of the regions involved in a hierarchical system responsible for organizing defensive behavior in both virtual and real image formats. 

**Figure 6:**
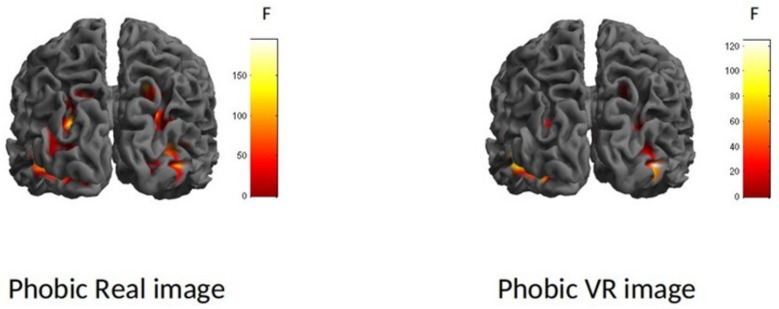
Visual activation of phobic stimuli processing (virtual and real format) in an adult sample with a specific phobia.

Finally, to test the research objective related to the functional processing of Wave2 with virtual or real image phobic stimuli, new ROI analyses were performed: We explored the involvement of visual and limbic brain areas and their connectivity, selecting the Brodmann visual area and the amygdala for study. The insula was also selected because of its functional relationship in processing interceptive inputs. Figure 6 shows the results obtained. Initial data showed that visual processing of the stimuli started similarly for both groups. The primary visual activity associated with phobic stimulus processing (BA17) was similar in the RI and VR groups. However, there were differences in the associative visual cortex (BA18 and BA19). Specifically, the real image was significant in a cluster (46, −72, −6) of the right occipital cortex with very extensive (52 BA18 voxels and 59 BA19 voxels) and intense activity (BA18 t mean = 4.75; t SD = 2.85 and BA19 t mean = 5.72; t SD = 3.82). In the VR group, significant activity occurred in both right and left clusters. In the right cluster (46, −72, −6), it was observed in the same coordinate with similar intensity (BA18 t mean = 5.71; t SD = 2.02 and BA19 t mean = 4.90; t SD = 3.33), but was less extensive (4 BA18 voxels and 11 BA19 voxels). In the left cluster (−30, −88, −18), significant activity was observed in the associative visual areas with the same extension in BA18 and BA19 (15 voxels) and stronger intensity (BA18 t mean = 5.51; t SD = 2.96 and BA19 t mean = 4.44; t SD = 1.80). These results suggested differential Wave2 phobic stimulus processing according to the image format.

## 4. Discussion

In the present study, we attempted to test whether exposure to virtual phobic stimuli activated the same brain areas as exposure to real image stimuli in a group of patients with phobias to small animals. This was explored specifically in regions usually associated with phobic stimulus processing: Amygdala, hippocampus, insula, fusiform gyrus, occipital cortex, calcarine area, and thalamus.

As pointed out by previous systematic reviews and meta-analyses, greater activations were found in the areas traditionally involved in fear processing [16,17,18,19,20]. These activations were found regardless of whether participants were exposed to virtual stimuli or real image stimuli. The absence of a main effect of image format supports the idea that virtual reality activates similar brain areas to those that real images do, including the regions involved in both Wave1 and Wave2 phobic stimulus processing. Main differences between VR and RI phobic stimuli were observed in activation intensity: Real images generated greater intensity in the hemodynamic response than virtual images. These data support an idea that is simple, but has clear implications for exposure technique: Real phobic images produce higher anxiety responses, but virtual stimuli also produce significant levels of anxiety [32]. These results can be understood as an initial endorsement of the use of VR as an exposure procedure [8] and not as a mere training opportunity [11,12].

As mentioned above, both VR and RI activated the two fear processing routes, but greater activation of the visual areas (inferior, medial, and superior occipital cortex and calcarine area) was observed with RI stimuli. The differences between real and virtual images were not found in the primary visual area (BA17), but in the associative visual areas (BA18 and BA19). The calcarine area has been found to play a main role in the visual network associated with the conditioning of fear [33,34]. These data indicate a greater activation of the Wave2/conscious route when participants are exposed to real image phobic stimuli. This greater involvement of associative visual processing has also been observed when the stimulus resembles the experience of the individual, as happens with in vivo exposure to the phobic stimulus [35,36]. These results support the idea that real images activate more areas from the Wave2 processing of phobic stimuli than VR and could be a therapeutic resource. Brain activation with feared stimuli produces higher levels of distress, and maintaining exposure to real images eventually starts to reduce anxiety. Consequently, the more patients become accustomed to facing the feared stimulus, the less anxious they will be when they face it again and, therefore, the less they will associate it with the expected negative response.

The study of the interaction between the independent variables in the various brain areas selected for their involvement in phobia processing, as shown by the ANOVA, revealed differences in the brain activation of cortical perceptual regions, but not in the limbic system regions. However, the differences in the ROI analysis observed in the amygdala and insula indicate that fear-related stimuli produced higher and more extensive brain activation when real images were processed compared to images presented in virtual reality. As shown by the data, there was also a greater activation (and extension) in the left insula when participants were exposed to RI compared to VR stimuli. It has been proposed that the insula integrates bottom-up interoceptive signals with top-down information [37]. Specifically, a signal is activated when affective visual stimuli are processed and this signal is guided by certain top-down requirements. The presence of feared stimuli represents an excellent opportunity for these requirements. Subsequently, this signal can be conveyed to control regions such as the prefrontal cortex for appropriate behavioral output. This “appropriate” behavior may take the form of an escape behavior for patients with phobias. In fact, the higher intensity of insula activation during real phobic stimulus presentation can be associated with more escape behaviors [38]. In addition, VR stimuli may be more suitable for the exposure procedure because they are less likely to activate escape behaviors.

As virtual stimuli affect ROIs that are usually related to brain responses to phobias, VR produces a significant subjective experience and generates a sense of presence. Thus, virtual phobic stimuli can produce a significant level of anxiety. Yet, as shown by the differences in brain activation, the immersive properties of virtual stimuli are lower than those of real images, but VR also activates a complex neural connectivity, including several associative areas, far from a mere activation of fear circuitry. Does this imply that VR can initiate an embodied simulation, aside from a rigorous representation of fear stimuli? As cited [15], embodied simulation is being proposed as the key mechanism for why VRET is effective, because VR provides a mental internal model, as a predictive coding regulating the body in its context effectively. As a result, more thorough experimental designs (with precise brain measures regarding embodiment) are needed. 

Meanwhile, in practice, exposure to real phobic images produces higher activations and it also may require a greater effort to voluntarily inhibit emotional activation than exposure to virtual stimuli and consequently may lead to more escape behaviors. Although there are no conclusive data on this [39], given that VR phobic stimulus exposure activates fewer escape behaviors, lower attrition rates and more therapeutic adherence can be expected, as reported by other studies [7,9,39,40]. For this, according to our data, virtual phobic stimuli require two paradoxical properties: They must be as similar as possible to real stimuli to activate the mechanisms associated with fear responses, but at the same time, participants need to be able to identify them as virtual. 

This study has several limitations. First, the small sample size may have affected the reliability of the results [41]. In addition, also due to sample size, sex differences could not be taken into account. Furthermore, this study only used one type of specific phobia (i.e., small animal phobia) with few experimental stimulation conditions: Data about comorbid phobias and evolution time were not taken into account, and these data can affect results. Moreover, we did not assess participants’ level of disgust as an emotional state different to fear/phobia [42], nor did we measure their escape behavior. However, the role of neural activity with escape/avoidance behavior is well established [43]. To add, we used 3D filmed real phobic stimuli as a representative condition to in vivo exposure, but this equivalence was not tested. Finally, we did not establish if the results were due to a phobic condition or could also be observed in anxious nonphobic individuals.

## 5. Conclusions

In short, the small animal images filmed in both real image and virtual reality formats generated the functional activation of the brain regions involved in the emotional processing of fear—thalamus, amygdala, hippocampus, fusiform gyrus, insula and occipital cingulate, and prefrontal cortices—in phobic individuals. However, real images produced more intense brain activations and a different pattern of hemodynamic responses than those elicited by virtual reality stimuli. This notwithstanding, these differences do not preclude the use of VR as an exposure resource, as the virtual images provided a sufficiently intense distress response in phobic individuals, activated a conscious process pathway, and, furthermore, led to fewer escape behaviors. These data support the use of virtual reality as an exposure procedure in the treatment of phobia disorders with similar properties to activate underlying mechanisms of exposure techniques.

## Figures and Tables

**Table 1:** fMRI comparisons between the RI group and the VR group in brain regions of interest (ROIs).

AREA	Coordinates	Hemisphere	*K*	*Z*	*P*
**Real Image > VR Phobics**					
Amygdala		Right/Left			n.s.
Hippocampus	22, −28, −6	Right	4	3.4	0.0001
Insula		Right/Left			n.s.
Fusiform gyrus	38, −72, −18	Right	11	4.54	0.0001
	22, −64, 14	Right	3	4.51	0.0001
	−34, -52, −22	Left	18	3.92	0.0001
	−26, −68, −6	Left	-	3.51	0.0001
	34, −48, −14	Right	4	3.37	0.0001
**Occipital cortex**					
Inferior		Right			n.s.
Middle	26, −88, 6	Right	49	4.29	0.0001
Superior	26, −72,34	Right	28	4.42	0.0001
Inferior		Left			n.s.
Middle	−30, −72, 26	Left	38	4.47	0.0001
Superior		Left			n.s.
Calcarine area	−14, −76, 1	Left	10	3.7	0.0001
Thalamus		Right/Left			n.s.

n.s.: Not significant. RI: Real Image; VR: Virtual Reality; AREA: Brain region, K: Voxel’s number, Z: Tipical score.
